# Prognostic factors in men with metastatic castration-resistant prostate cancer treated with cabazitaxel

**DOI:** 10.18632/oncotarget.22474

**Published:** 2017-11-16

**Authors:** Bodine P.S. Belderbos, Ronald de Wit, Esther Oomen-de Hoop, Annemieke Nieuweboer, Paul Hamberg, Robbert J. van Alphen, André Bergman, Nelly van der Meer, Sander Bins, Ron H.J. Mathijssen, Robert J. van Soest

**Affiliations:** ^1^ Department of Medical Oncology, Erasmus University Medical Center and Cancer Institute, 3015 CE Rotterdam, The Netherlands; ^2^ Department of Internal Medicine, Franciscus Gasthuis and Vlietland, 3045 PM Rotterdam, The Netherlands; ^3^ Department of Internal Medicine, Elisabeth Tweesteden Ziekenhuis, 5042 AD Tilburg, The Netherlands; ^4^ Department of Medical Oncology, Netherlands Cancer Institute–Antoni van Leeuwenhoek, 1066 CX Amsterdam, The Netherlands; ^5^ Department of Urology, Erasmus University Medical Center and Cancer Institute, 3015 CE Rotterdam, The Netherlands

**Keywords:** prognostic factors, cabazitaxel, mCRPC, overall survival, PSA response

## Abstract

**Background:**

Treatment selection for men with metastatic castration-resistant prostate cancer (mCRPC) has become increasingly challenging with the introduction of novel therapies at earlier disease stages. The purpose of this study was to identify prognostic factors for overall survival (OS) and PSA response in patients with mCRPC treated with cabazitaxel.

**Results:**

224 mCRPC patients were included in the current analysis. In multivariable analysis, WHO performance status, baseline hemoglobin, alkaline phosphatase and albumin were all significantly associated with OS. Hemoglobin and alkaline phosphatase were significantly associated with PSA response.

**Conclusions:**

This study identified prognostic factors for OS and PSA response of men with mCRPC treated with cabazitaxel. In an increasingly complicated treatment landscape with several treatment options available our findings might serve to estimate the chance of survival of men qualifying for treatment with second-line chemotherapy in daily practice. Furthermore, these data can be used to risk-stratify patients in clinical trials.

**Methods:**

We performed a post-hoc analysis of a randomized phase II trial of mCRPC patients treated with cabazitaxel. Cox and logistic regression models were used to investigate the influence of clinical and biochemical variables on OS and PSA response. Nomograms were developed to estimate the chance of PSA response and OS.

## INTRODUCTION

With the introduction of novel agents, treatment options for metastatic castration-resistant prostate cancer (mCRPC) have notably evolved in the past few years. Since level one evidence supporting an optimal treatment sequence in mCRPC is lacking, the choice of treatment by the clinician has become increasingly challenging. Treatment decisions are generally made on the basis of clinical symptoms, comorbidities, expected side-effects and preferences by the patient and the treating physician. Risk classification and prognosis assessment remain critical before the start of a new therapy. Therefore, it is important to identify biomarkers, predictive and prognostic factors for clinical outcome, in order to simplify treatment choice and timing. Such factors may serve to predict individual prognosis at the start of therapy and to classify patients in different risk-groups, which can also be used for stratification in clinical trials.

Prognostic models and nomograms for mCRPC patients receiving first-line chemotherapy have been developed based on large phase III trials [1–3], including parameters such as performance status, time since last docetaxel use, the presence of measurable disease, the presence of visceral disease, the presence of pain, duration of hormonal treatment, hemoglobin (Hb), prostate specific antigen (PSA) and alkaline phosphatase (AP). For second-line chemotherapy with cabazitaxel, prognostic factors were identified based on the TROPIC trial [4, 5]. However, these findings have not been investigated in other datasets of men treated with cabazitaxel. In the current study, we aimed to identify prognostic factors for men with mCRPC receiving cabazitaxel. For this purpose, we used data from a multicenter randomized phase II trial [6].

## RESULTS

### Patient characteristics

Baseline characteristics were available for 224 patients and are shown in Table 1. The characteristics were similar to other studies of men treated with second-line chemotherapy [5].

**Table 1:** Patients characteristics

Characteristic	Value No. (%)
All	224 (100)
Age, mean ± SD	68.8 ± 7.2
WHO	
0	92 (41)
1	130 (58)
Missing	2 (1)
Type castration	
Surgical	30 (13)
LHRH-analogue	194 (87)
No. prior therapies	
1	204 (91)
≥2	20 (9)
Months since last chemotherapy	
≤6 months	102 (46)
>6 months	122 (54)
**Baseline laboratory results**	**Median (IQR)**
Hb (mmol/L)	7.7 (6.8–8.2)
Albumin (g/L)	39.0 (36.0–43.0)
AP (U/L)	130.0 (86.0–262.0)
LDH (U/L)	271.0 (210.0–392.0)
ANC (10^9^/L)	5.9 (4.4–7.6)
PSA (g/L)	154.1 (59–388.3)
dNLR	2.7 (1.8–3.9)

Abbreviations: WHO = world health organization, Hb = hemoglobin, AP = Alkaline Phosphatase, LDH = lactate dehydrogenase, ANC = absolute neutrophil count, PSA = prostate specific antigen, dNLR = derived Neutrophil-Lymphocyte ratio.

### OS

Median OS of patients in this dataset was 13.3 months (IQR 7.0–22.3). Results of the univariable and multivariable analyses for OS are shown in Table 2. Time since last chemotherapy, neutropenia grade 3/4, PSA and LDH were significantly associated with OS in univariable analysis, but not in multivariable analysis. In multivariable analysis, four parameters remained significantly associated with OS; WHO performance status (HR 1.49, 95% CI 1.04–2.13, *p* = 0.028), Hb (HR 0.73, 95% CI 0.61–0.87, *p* = 0.001), AP (HR 1.50, 95% CI 1.18–1.91, *p* = 0.001) and Alb (HR 0.92, 95% CI 0.89–0.95, *p* < 0.001). Of note, dNLR (*p* = 0.45) was not associated with OS in univariable analysis.

**Table 2:** Univariable and multivariable model for OS

Variable	Univariable HR (95% CI)	*P*-value	Multivariable HR (95% CI)	*P*-value
WHO PS	1.57 (1.16–2.13)	0.004	1.49 (1.04–2.13)	0.028 (1 vs 0)
Time since last therapy (>6 mths)	0.59 (0.44–0.79)	<0.001		
Neutropenia (Gr.3/4)	0.67 (0.50–0.90)	0.007		
Hb (mmol/L)	0.57 (0.49–0.67)	<0.001	0.73 (0.61–0.87)	0.001
PSA (log, g/L)	1.12 (1.01–1.26)	0.040		
AP (log, U/L)	1.83 (1.49–2.25)	<0.001	1.50 (1.18–1.19)	0.001
LDH (log, U/L)	2.37 (1.73–3.25)	<0.001		
Albumin (g/L)	0.91 (0.89–0.94)	<0.001	0.92 (0.89–0.95)	<0.001

Univariable *N* = 206–224 (because of missing values the number of included patients can differ per variable) Multivariable *N* = 200.

Abbreviations: WHO PS = world health organization performance status, Hb = hemoglobin, PSA = prostate specific antigen, AP = alkaline phosphatase, LDH = lactate dehydrogenase, log = log transformed variables when data were not normally distributed.

**Figure 1:**
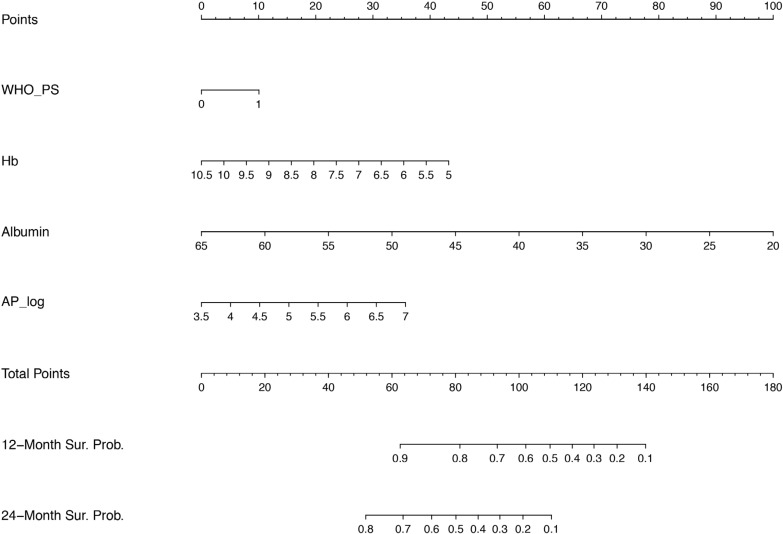
Nomogram for OS Prognostic nomogram predicting overall survival probability. For each variable, starting with WHO performance score on the second axis, draw a vertical line up to the ‘Points’ axis (top line) to identify the number of prognostic points the patient receives for the value of this variable. Calculate the ‘Total Points’ by adding up the prognostic points for each variable. Determine the 12 month or 24 month overall survival by drawing a vertical line from the ‘Total Points’ axis down to the axis indicating the survival probabilty. WHO PS = world health organization performance status, Hb = hemoglobin, AP = alkaline phosphatase, log = log transformed variables when data were not normally distributed.

Figure 1 presents a nomogram that is based on the multivariable model for OS. This nomogram can be used to predict the individual survival probability at 12 and 24 months. For example, a patient with a performance score of 1, an Hb level of 7.0 mmol/L, albumin level of 40 g/L and AP of 90 U/L (log_A*P* = 4,5) has a 12 month survival probability of approximately 60% and a 24 month survival probability of approximately 20%.

### PSA response

**Figure 2:**
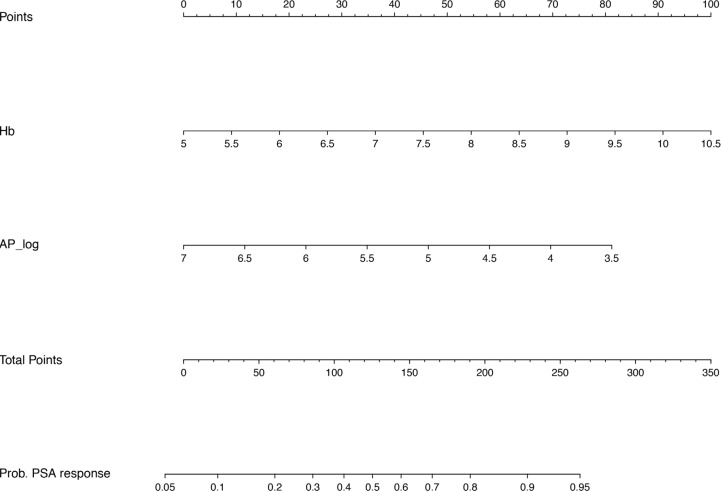
Nomogram for PSA response Prognostic nomogram predicting PSA response. For both variables, Hb and AP_log, draw a vertical line from the absolute value of this variable up to the ‘Points’ axis (top line) to identify the number of prognostic points the patient receives for these variables. Calculate the ‘Total Points’ by adding up the prognostic points for both variables. Determine the probability of PSA response by drawing a vertival line form the ‘Total Points’ axis down to the axis of the probability of PSA response. Hb = hemoglobin, AP = alkaline phosphatase, log = log transformed variables when data were not normally distributed.

Univariable analysis showed significant associations between PSA response and WHO performance status, Hb, AP and LDH. In the multivariable model Hb and AP remained significantly associated with PSA response and were taken into the final model (Table 3). Higher hemoglobin level before treatment (OR 1.48, 95% CI 1.05–2.07, *p* = 0.024), and a lower AP level at the start of treatment (OR 0.61, 95% CI 0.39–0.96, *p* = 0.034) resulted in a higher chance of PSA response. Figure 2 displays a nomogram to calculate the chance of PSA response for an individual patient treated with cabazitaxel based on our multivariable model.

**Table 3:** Univariable and multivariable model for PSA response

Variable	Univariable OR (95% CI)	*P*-value	Multivariable OR (95% CI)	*P*-value
WHO PS	0.48 (0.27–0.84)	0.011		
(1 vs 0)				
Hb (mmol/L)	1.67 (1.22–2.29)	0.002	1.48 (1.05–2.07)	0.024
AP (log, U/L)	0.50 (0.33–0.77)	0.002	0.61 (0.39–0.96)	0.034
LDH (log, U/L)	0.49 (0.27–0.88)	0.016		

Univariable *N* = 217–224 (because of missing values the number of included patients can differ per variable) Multivariable *N* = 215.

Abbreviations: WHO PS = world health organization performance status, Hb = hemoglobin, A*P* = alkaline phosphatase, LDH = lactate dehydrogenase, log = log transformed variables when data were not normally distributed.

## DISCUSSION

In this post-hoc analysis of a large phase II trial of patients with mCRPC treated with cabazitaxel, we found that WHO performance status, baseline Hb, AP and albumin were all significantly associated with OS. In addition, Hb and AP were identified as parameters to predict the probability of PSA response in mCRPC patients receiving second-line chemotherapy. To our knowledge, we are the first to report prognostic factors and a nomogram for OS and PSA response in men with mCRPC treated with cabazitaxel outside of the TROPIC registration trial [5]. Treatment selection of men with metastatic prostate cancer has become increasingly challenging with the introduction of novel therapies at earlier disease stages. In this changing treatment paradigm, predictors and prognostic factors of treatment response and outcome are still lacking. The current study shows that readily available, cheap and easy to use clinical parameters are of prognostic value for estimating the chance of PSA response and OS using the presented nomograms (Figures 1 and 2). These individual probabilities of survival and response might help the physician to decide when to initiate treatment in daily clinical practice. As an illustration, if the identified prognostic factors follow a trend towards an unfavourable condition, it may help the physician to avoid missing the window of opportunity for cabazitaxel treatment and initiate therapy before the patients’ condition is declining.

Several distinct predictors of OS and PSA response (e.g. albumin) were identified as compared to the models constructed from the TROPIC dataset [5, 7]. The difference between the identified variables in the final model from Halabi et al. and our cohort may be due to a different population, different primary trial design and different model assumptions. The strength of our model is that it is based only on men treated with cabazitaxel. In contrast, the Halabi nomogram has been constructed based on the TROPIC trial and validated on the SPARC trial, including also men treated with mitoxantrone and satraplatin which are not used in the current treatment armamentarium [5]. Therefore, our study might represent a more contemporary real-world patient population. This study is limited by its post-hoc design. As a result, radiographic variables such as the presence of visceral disease were not collected in the primary trial.

dNLR, a parameter to determine the inflammatory response rate of the host, has been reported and validated as a prognostic factor for OS and response in mCRPC patients treated with docetaxel, cabazitaxel and abiraterone [7–10]. In addition, a high dNLR was associated with poor OS in other tumor types [11]. However, in our analyses no significant association between dNLR and OS, nor between dNLR and PSA response was found. Furthermore, in a post-hoc analysis of the TROPIC trial neutropenia during treatment with cabazitaxel has shown a significant relation with OS of men with mCRPC [12]. Although we had comparable treatment groups, no association between the occurrence of grade 3–4 neutropenia and OS or PSA response was found.

In conclusion, this study identified clinical and biochemical variables associated with OS and PSA response of patients with mCRPC treated with cabazitaxel. In an increasingly complicated treatment landscape with several treatment options available including chemotherapy and novel AR-targeted agents, our findings have prognostic value for treatment response and survival of men qualifying for treatment with second-line chemotherapy in daily practice. Furthermore, these data can be used to risk-stratify patients in future clinical trials.

## PATIENTS AND METHODS

### Patients

We performed a post-hoc analysis of a randomized phase II trial (CABARESC, NTR2991). The CABARESC trial was designed to investigate the influence of budesonide (9 mg daily) on cabazitaxel pharmacokinetics and cabazitaxel-induced diarrhea and was reported elsewhere [6]. Between December 2011 and October 2015, a total of 246 mCRPC patients were included in the study. The study was conducted in 22 Dutch hospitals and was approved by the ethics committee of the Erasmus MC (MEC 11-324) and all local institutional review boards. Written informed consent was obtained from all participants.

Full study details are described in the original paper [6]. Briefly, patients were eligible if they had metastatic castration-resistant adenocarcinoma of the prostate with disease progression during or after docetaxel therapy, defined as two consecutive rises in PSA (taken ≥1 week apart) or according to RECIST criteria. Full inclusion criteria are shown in Supplementary materials. In this study, no significant impact of budesonide on the pharmacokinetics of cabazitaxel and cabazitaxel-induced diarrhea was found. We have previously shown that there was no influence of prior treatment with abiraterone acetate or enzalutamide on OS and PSA response of men treated with cabazitaxel [13]. Patients who were deemed inevaluable in the original CABARESC trial (*N* = 19) (Supplementary Table 1) and patients with missing or inadequate PSA values were considered ineligible for the current analysis (*N* = 3), leaving 224 patients evaluable for the current analysis.

### Biomarker panel

From all evaluable patients, laboratory and clinical factors collected at baseline and during treatment were available. The biochemical parameters were collected at baseline included: PSA, lactate dehydrogenase (LDH), AP, albumin (Alb), Hb, derived neutrophil-lymphocyte ratio (dNLR), WHO performance status (0 vs 1), age, type of castration (GnRH analogues vs. surgical castration) and time since last chemotherapy cycle (>6 months vs ≤6 months). The dNLR was computed by the absolute neutrophil count (ANC) divided by the absolute white blood cell count (WBC) minus the ANC, (ANC/ (WBC-ANC)). The occurrence of ≥grade 3 neutropenia during treatment was collected. Log transformation was applied to variables with a non-normal distribution.

### Primary objective and definitions

The objective of the current analysis was to identify prognostic factors associated with OS and PSA response in mCRPC patients treated with cabazitaxel. OS was defined by time from randomization to death from any cause. Patients still alive at the end of the study were censored. Prostate Cancer Working Group 2 (PCWG2) criteria were used to define PSA response as a ≥50% decline from baseline measured twice 3 to 4 weeks apart [14].

### Statistical analysis

Descriptive statistics were used to summarize patients’ characteristics. Univariable and multivariable Cox regression analyses were used to investigate the influence of laboratory and clinical parameters on OS. Univariable and multivariable logistic regression analysis were used with PSA response (≥50%) as the dependent variable and baseline parameters were included as covariates. Factors with a *p* < 0.10 in univariable analysis were entered into the multivariable analysis. The multivariable model was constructed using backward selection at the 5% level. Data were analyzed using STATA^®^ version 14 (Stata-Corp LP TX, USA).

Based on the multivariable model for OS and PSA response we have generated a nomogram for OS and PSA response. Software program ‘R’ was used to generate both nomograms.

## SUPPLEMENTARY MATERIALS TABLE



## Abbrevations

ANC: absolute neutrophil count
AP: alkaline phosphatase
Alb: albumin
CI: confindence interval
dNLR: derived neutrophil-lymphocyte ratio
GnRH: Gonadotropin releasing hormone
Hb: hemoglobin
HR: hazard ratio
IQR: inter quartile range
LDH: lactate dehydrogenase
mCRPC: metastatic castration resistant prostate cancer
OR: odds ratio
OS: Overall survival
PCWG2: prostate cancer working group 2
PSA: prostate specific antigen
RECIST: response evaluation criteria in solid tumor
WBC: white blood cell count
WHO PS: world health organization performance status
