# Supplementary material for: Prognostic factors in men with metastatic castration-resistant prostate cancer treated with cabazitaxel
Source: Oncotarget. 2017 Nov 16;8(63):106468–74. doi: 10.18632/oncotarget.22474 (PMC5739748; doi:10.18632/oncotarget.22474)
Supplement: Supplementary file 1 [file oncotarget-08-106468-s001.pdf]

Prognostic factors in men with metastatic castration-resistant prostate cancer treated with cabazitaxel

SUPPLEMENTARY MATERIALS

Inclusion and exclusion criteria CABARESC study:

Inclusion criteria

- Metastatic castrate resistant prostate cancer (mCRPC) patients with documented disease progression, defined as: documented rising PSA levels (at least 2 consecutive rises in PSA over a reference value taken at least 1 week apart, or a PSA rise of ≥2.0 µg/l), appearance of new lesions or documented disease progression based on CT scan or bone scan.
- Previous treatment with a docetaxel-containing regimen
- Age ≥ 18 years;
- WHO performance status ≤1
- Adequate renal function (within 21 days before randomization) defined as serum creatinin ≤1.5 × ULN and/or calculated creatinin clearance ≥50 ml/min, according to MDRD formula.
- Adequate hepatic functions (within 21 days before randomization) defined as: total bilirubin ≤1.0 × ULN; alanine aminotransferase (ALT) and aspartate aminotransferase (AST) ≤2.5 × ULN, in case of liver metastasis <5 × ULN; alkaline phosphatase (AP) <5 × ULN) In case of bone metastasis, AP <10 × ULN is accepted.
- Adequate hematological blood counts (within 21 days before randomization) defined as (absolute neutrophil count (ANC) ≥1.5 × 10^9/L and platelets ≥100 × 10^9/L);
- Castration, either surgically or by continued LHRH agonist therapy
- Written informed consent according to ICH-GCP

Exclusion criteria

- Impossibility or unwillingness to take oral drugs;
- Serious illness or medical unstable condition requiring treatment, brain metastases or history of psychiatric disorder that would prohibit the understanding and giving of informed consent.
- Use of medications or dietary supplements known to induce or inhibit CYP3A
- Known hypersensitivity to corticosteroids
- Any active systemic or local bacterial, viral, fungal–or yeast infection.
- Ulcerative colitis, Crohn's disease or celiac disease (active or in medical history)
- Ostomy
- Planned/active simultaneous yellow fever vaccine
- Geographical, psychological or other non-medical conditions interfering with follow-up

Supplementary Table 1: Reasons for exclusion

Reasons for exclusion	Group CABA	Group BUD	Post-hoc	Total
No cabazitaxel (in study context)*	3	7	0	10
Initial cabazitaxel dose <25mg/m^2	5	1	0	6
Treatment started before randomization	1	0	0	1
Death before start therapy	1	0	0	1
Long treatment delay after randomization^	1	0	0	1
Missing laboratory values	0	0	3	3
**Total**	11	8	3	22

*due to disease progression and worsening of patient conditions: patient did not receive treatment or not in context of this study. ^due to ASAT and ALAT >2 upper limit of normal without liver metastases there was a time of two months between randomization and start of cabazitaxel therapy.
